# Letter to the editor: The impact of surgeons’ visual angle misperception on acetabular cup positioning accuracy: a retrospective multicenter cohort study

**DOI:** 10.1097/MS9.0000000000004318

**Published:** 2025-11-24

**Authors:** Yi Zhang, Xiguang Zhang, Qiaoning Yue

**Affiliations:** a Department of Orthopedics, The People’s Hospital of Yuxi City, The Sixth Affiliated Hospital of Kunming Medical University, Yuxi, Yunnan, China; b Department of Graduate School, Kunming Medical University, Kunming, Yunnan, China

We commend Hu *et al* for spotlighting a human-factors mechanism – surgeons’ visual angle misperception – that may systematically shift acetabular cup orientation during total hip arthroplasty^[1]^. By integrating a visual task, a benchtop platform, and a multicenter cohort, the authors show that experience narrows variability without abolishing bias. For transparency, this correspondence was prepared in line with the TITAN guideline on artificial intelligence-assisted writing, with human oversight throughout; no generative content beyond language assistance was introduced^[2]^.

From a measurement standpoint, aligning intraoperative targets and postoperative readouts within a common reference frame would enhance clarity. Radiographic, operative, and anatomic definitions are not interchangeable; without explicit conversion rules (e.g., anatomical positioning protocol [APP]/functional orientation), method variance can masquerade as perceptual error^[3,4]^. Providing a compact schematic that maps definitions, coordinate systems, and operative-to-radiographic transformations would substantially improve interpretability.

Regarding orientation estimation, EBRA’s strength lies in migration/wear tracking, and its accuracy for absolute anteversion/inclination depends on pelvic pose, beam geometry, and rim visualization. Adding inter-reader agreement (intraclass correlation coefficient with 95% confidence intervals) and Bland–Altman plots versus a computed tomography-based or validated geometric method would quantify systematic offsets and limits of agreement beyond mean comparisons^[5,6]^. Specifying minimal acceptable limits for agreement would further support auditability.

For bench-to-bedside inference, vantage- and approach-dependent asymmetry is plausible (right-handed surgeons, posterolateral approach, lateral decubitus). Re-estimating key effects with a mixed-effects specification that nests hips within surgeons and centers – with side and approach as fixed effects – would separate viewpoint effects from misperception while addressing cluster imbalance. This modeling step would also clarify whether the observed bias persists after adjusting for case-mix and exposure.

In terms of clinical translation, reliance on legacy “safe zones” risks oversimplification. Dislocations can occur within classical zones, and functional posture plus hip–spine coupling modulate effective cup orientation^[7–9]^. Sensitivity analyses under a functional safe-zone framework and, where outcomes are available, expression of angular deviations as absolute risk differences or numbers-needed-to-treat at clinically used thresholds would make decisions more transparent across centers.

To emphasize clinical interpretability, reporting the smallest detectable change and equivalence margins for anteversion/inclination, alongside calibration-in-the-large and per-surgeon calibration slopes with 95% prediction intervals, would show that experience reduces scatter while any systematic bias is explicitly quantified. Where platforms share a manufacturer with the robotic reference, a concise statement of roles, calibration workflow, and any intellectual-property/financial interests would align with best practice without detracting from the central message.

In summary, by harmonizing angle definitions, quantifying agreement with appropriate metrics, modeling clustered data with side/approach terms, and situating findings within a functional, outcome-aware framework, the work can further strengthen its translational value while preserving its central insight: experience reduces scatter more than it removes systematic bias.

## Data Availability

The data used in this study are available upon reasonable request to the corresponding author. Due to privacy restrictions, some datasets may require additional ethical clearance before sharing. No patient-level information was used in this Correspondence; our remarks are based solely on publicly available sources, including the study on surgeons’ visual-angle misperception in THA. As no de-identified data, code, or images were created, repository submission is not required. Requests for materials should be directed to the primary report authors: Hu Y, Jin M, Qi Y, et al. Int J Surg. Publish Ahead of Print. doi: 10.1097/JS9.0000000000003429.
